# Assessing Patient-Perceived Hospital Service Quality and Sentiment in Malaysian Public Hospitals Using Machine Learning and Facebook Reviews

**DOI:** 10.3390/ijerph18189912

**Published:** 2021-09-21

**Authors:** Afiq Izzudin A. Rahim, Mohd Ismail Ibrahim, Kamarul Imran Musa, Sook-Ling Chua, Najib Majdi Yaacob

**Affiliations:** 1 Department of Community Medicine, School of Medical Science, Universiti Sains Malaysia, Kubang Kerian, Kota Bharu 16150, Kelantan, Malaysia; drafiqrahim@student.usm.my (A.I.A.R.); drkamarul@usm.my (K.I.M.); 2 Faculty of Computing and Informatics, Multimedia University, Persiaran Multimedia, Cyberjaya 63100, Selangor, Malaysia; slchua@mmu.edu.my; 3 Units of Biostatistics and Research Methodology, School of Medical Sciences, Health Campus, Universiti Sains Malaysia, Kubang Kerian, Kota Bharu 16150, Kelantan, Malaysia; najibmy@usm.my

**Keywords:** machine learning, social media, Facebook, service quality, SERVQUAL, sentiment, patient online review, accreditation, Malaysia

## Abstract

Social media is emerging as a new avenue for hospitals and patients to solicit input on the quality of care. However, social media data is unstructured and enormous in volume. Moreover, no empirical research on the use of social media data and perceived hospital quality of care based on patient online reviews has been performed in Malaysia. The purpose of this study was to investigate the determinants of positive sentiment expressed in hospital Facebook reviews in Malaysia, as well as the association between hospital accreditation and sentiments expressed in Facebook reviews. From 2017 to 2019, we retrieved comments from 48 official public hospitals’ Facebook pages. We used machine learning to build a sentiment analyzer and service quality (SERVQUAL) classifier that automatically classifies the sentiment and SERVQUAL dimensions. We utilized logistic regression analysis to determine our goals. We evaluated a total of 1852 reviews and our machine learning sentiment analyzer detected 72.1% of positive reviews and 27.9% of negative reviews. We classified 240 reviews as tangible, 1257 reviews as trustworthy, 125 reviews as responsive, 356 reviews as assurance, and 1174 reviews as empathy using our machine learning SERVQUAL classifier. After adjusting for hospital characteristics, all SERVQUAL dimensions except Tangible were associated with positive sentiment. However, no significant relationship between hospital accreditation and online sentiment was discovered. Facebook reviews powered by machine learning algorithms provide valuable, real-time data that may be missed by traditional hospital quality assessments. Additionally, online patient reviews offer a hitherto untapped indication of quality that may benefit all healthcare stakeholders. Our results confirm prior studies and support the use of Facebook reviews as an adjunct method for assessing the quality of hospital services in Malaysia.

## 1. Introduction

The patient-centered approach (PCA) has become a critical component in the development and enhancement of health services and patient care. It values the important input of medical consumers in order to develop aspects of healthcare services that improve patients’ and consumers’ experiences. Consumers and patients have been more involved in talks among stakeholders and health care task groups in recent years. Nonetheless, with the goal of actively including health consumers in the transformation and reconstruction of quality care activities, debate persists about whether PCA methods should be adopted or if conventional organizational requirements seem to take precedence [1]. Over the past decade, quality management studies have emphasized PCA as a critical component of high-quality care delivery [2,3,4]. Patients may be the most trustworthy journalists when it comes to some aspects of the health care process; their perspectives should be taken into account when advocating for reforms to enhance patient safety [5]. The Scottish Health Agency is an example of a healthcare organization that has changed its emphasis to a patient-centered approach. Their health and social care policies have shifted in recent years from a hierarchical approach centered on hospitals to an integrated, co-management, and community-based approach [6].

The balance between patient demands and quality improvement programs is critical, as it influences patient safety, life and death, and long-term health [7]. A systematic analysis concluded that poor healthcare quality was the primary factor contributing to an increase in fatalities from cardiovascular disease, newborn traumas, and communicable diseases [8]. As healthcare prepares for the effect of Industrial Revolution 4.0 by becoming more patient-centered and value-driven, quality management programs must include efforts that identify and respect patients’ interests, wants, and beliefs. Because such reports can only be produced by patients, it is essential to establish mechanisms to monitor patient experiences and to encourage their usage at both the individual and community level [9,10]. Furthermore, by eliciting and enhancing patient perceptions of treatment quality via PCA methods, the likelihood of medical, medication, and laboratory mistakes will be reduced [2].

Structured patient satisfaction and quality measure surveys, such as the Hospital Consumer Assessment of Healthcare Providers and Systems (HCAHPS) and Service Quality (SERVQUAL) questionnaires, are often used to assess healthcare quality services [11,12,13,14]. SERVQUAL, HCAHPS, and other standard quality assessments are the product of years of evaluative analysis, are conducted and evaluated in a systematic manner, and can capture a significant number of patient answers per institution [14,15,16]. However, traditional patient or public surveys used to evaluate the quality of healthcare services are time and resource-intensive, requiring significant time between hospital admission and report disclosure, frequently resulting in a failure to identify the underlying causes of concerns, and possibly introducing response and selection bias [11,17]. Meanwhile, healthcare authorities now have an alternative to conventional patient surveys through social media [3]. There is increasing awareness that user-generated material available through social media platforms such as Facebook, Twitter, and Yelp may be utilized as a rich source of data for patient experience and quality-of-care metrics [11,12,18]. By improving their early-warning capabilities for healthcare quality management, such data may be utilized to augment and expand the breadth of patient experience and health quality services [19].

Numerous studies believe that social media represents the next horizon for provider-patient communication in healthcare. In Malaysia, Facebook is extensively utilized, and its market share continues to rise and in 2020, Facebook was the country’s most popular social networking site [20]. Facebook reviews is a technology that allows people to provide narrative reviews on organizations’ Facebook pages, and the feature offers insight into how the public perceives healthcare services [21]. Few studies have been conducted in the past to evaluate Facebook reviews of hospital services and nursing homes and found a low to moderate correlation between Facebook ratings and patient satisfaction metrics from systematic surveys [18,22,23,24]. With an increasing number of patients seeking and publicly sharing hospital ratings and reviews on Facebook, data collected via the feedback channel may be objectively related to traditional patient satisfaction or quality measure surveys such as SERVQUAL, HCAHPS, Consumer Assessment of Healthcare Providers and Systems (CAHPS) Dental Plan Survey, hospital accreditation, and clinical outcomes [11,25,26,27].

Nonetheless, social media data are often massive in quantity, posing challenges such as data cleaning, data processing, and the creation of a well-established empirical model of social media content quality [28]. While this may be accomplished manually via human input, its validity and reliability are widely disputed. As a result, such obstacles may be addressed using trained machine learning algorithms for this approach. A machine learning method for evaluating sentiment and classifying service quality based on unstructured social media data has the potential to substantially enhance both patients’ and healthcare professionals’ diagnosis and treatment of a range of health-related problems [29,30,31].

The purpose of this paper is to ascertain the prevalence of SERVQUAL dimensions and sentiments in Facebook reviews of Malaysia public hospitals. Second, we want to decipher the determinants of positive sentiment in hospital Facebook reviews. Thirdly, we are interested in determining the relationship between hospital accreditation and sentiments expressed in hospital Facebook reviews. Our study contributed mainly:

- To develop a novel and systematic method for converting social media comments to SERVQUAL dimensions and analyzing online sentiments in Malaysia via supervised learning.
- To classify topics based on an established methodology for service quality; SERVQUAL that is extensively used to assess the quality of health care services, overcoming obstacles, and providing policymakers with precise action implications.
- By identify the determinants of positive sentiment as well as its relationship with hospital accreditation in Malaysia using advanced statistical analysis.
- Via real-time monitoring of hospital quality and patient perceptions of health care services through the translation of social media data.
- Through the machine learning technology that can be utilized as an early-warning system for immediate quality improvement in healthcare.

## 2. Literature Reviews

### 2.1. Social Media Data

Patients and the public are increasingly using the Internet to discuss their healthcare experiences and to compare doctors and treatments [32,33]. The digital consumer movement on social media influenced patient autonomy and self-determination in medical treatment, highlighting the essential importance of online patient experience in determining health care quality [3,34]. While many studies have examined the use of social media in hospital settings, the bulk of them examines the use of Twitter or Yelp as a social media tool for evaluating the quality of hospital services, rather than the Facebook platform [11,12,17,35]. This is very certainly due to a population’s preference for social media in various countries.

As is the case with other social media platforms, Facebook ratings provide insight into the public’s perception of healthcare services. Numerous studies have been conducted in the past to assess Facebook ratings for hospital services and found a weak to moderate correlation between Facebook ratings and patient satisfaction metrics from systematic surveys [36,37]. Additionally, a local study discovered a modest connection between hospital patient satisfaction surveys and online satisfaction in Facebook reviews [38]. Moreover, with an increasing number of patients seeking and publicly sharing hospital ratings and reviews on Facebook, data collected via the feedback channel may be objectively associated with other hospital quality measures such as accreditation, clinical outcome indicators, and patient safety goals [18,36,39]. Reduced readmission rates are associated with an increased probability of patients recommending the hospital and, ultimately, with better Facebook ratings, according to a Facebook study [39]. However, another research found no correlation between Facebook user ratings and the 30-day all-cause readmission rate or Medicare expenditure per beneficiary ratio [22]. Meanwhile, a local study found no correlation between online patient satisfaction as expressed in Facebook reviews and hospital accreditation [38]. 

### 2.2. SERVQUAL Dimensions

SERVQUAL is a commonly used quality assessment method for assessing service quality across a range of service settings, industries, and countries [40]. The approach enables the efficient quantification of both customer service needs and perceptions of customer service [41,42]. SERVQUAL’s scale development showed five aspects of perceived quality: tangibles, reliability, responsiveness, assurance, and empathy. The ‘tangibles’ dimension encompasses elements of the service quality experience that are physical in nature (e.g., equipment, facilities, personnel). The characteristics of ‘reliability’ and ‘assurance’ represent customers’ views of the service provider’s capacity to provide the service. The former entails evaluating the service provider’s capabilities in terms of reliability and accuracy, while the latter entails evaluating the service provider’s characteristics such as knowledge and courtesy, which may inspire trust and confidence in the provider. The ‘responsiveness’ component is concerned with the service provider’s perceived helpfulness and promptness. Finally, the ‘empathy’ component refers to how individuals perceive customized, caring service [42].

SERVQUAL dimensions have been used to assess the quality of service in hospital and healthcare settings, mainly via survey-based techniques. Several local studies have developed and validated a SERVQUAL model for assessing the quality of healthcare services in Malaysia [13,43,44,45]. SERVQUAL and other quality measures are the results of years of evaluation, are performed and assessed in a systematic way, and can collect many patient responses per institution [14,15]. Nonetheless, the surveys have several disadvantages, including being expensive to administer, time-consuming, requiring significant time between hospitalization and public publication of results, frequently failing to identify the underlying cause of reported problems, and being susceptible to selection and response bias [3,11,12,46]. The distinction between traditional patient surveys and real-time public opinion on healthcare services demonstrates the need for additional data sources for assessing real-time public opinion on healthcare services [47]. As a result, the internet and social media have been suggested as a new way for evaluating and monitoring the quality of healthcare services [21,46,48,49]. 

### 2.3. Automation of SERVQUAL and Sentiment Classification

Social media data is often enormous and poses a variety of challenges, including data cleaning, data processing, and the establishment of a theoretical model of social media content quality. While this may be conducted manually via human input, the process is time-consuming, and the method’s validity and reliability are often questioned. A systematic study of patient online reviews established and suggested the use of advanced analytical techniques such as machine learning to expedite the processing of large-scale online review data [3]. Additionally, the systematic review advocated for conducting an in-depth study on the content of online reviews rather than just comparing structured data to social media ratings. Monitoring service quality through hospital social media platforms may aid all stakeholders in identifying quality aspects and reducing the need for costly and time-consuming surveys. Despite their rarity, research on Facebook content analysis shows a correlation between quality domains in social media evaluations and conventional quality assessments [22,36,37,38].

The term “topics” or “text classification” refers to the act of categorizing a collection of textual texts according to their content. Machine learning allows automated subject analysis via the use of different algorithms, which fall primarily into two categories: supervised and unsupervised learning. The distinction between these two major groups is the presence of labels in the subset of training data [50]. Apart from the use of input characteristics, supervised machine learning entails the use of predefined output attributes. The algorithms try to forecast and classify the preset attribute, and their accuracy and misclassification, as well as other performance metrics, are based on the counts of the predetermined attribute that are properly predicted or classified or not correctly predicted or classed. Manual classification is a method that is often employed in supervised learning. Numerous studies have used this technique to ascertain the topics of discussion in online patient reviews [11,17,27,30,48,51,52,53,54,55,56,57,58].

Unsupervised learning, on the other hand, is pattern recognition without the use of a target characteristic. Unsupervised algorithms discover underlying groups in unlabeled data and then label each value. Topic modeling is a method for automatically detecting themes within a given comment, with Latent Dirichlet Allocation (LDA) being the most often used method. Several studies used the method to explore themes or topics of discussion in patient online reviews [12,52,59,60,61,62,63] or classified tweets using the SERVQUAL dimensions [64].

Another machine learning technique is semi- or partial-supervised learning, which builds classifiers using mostly unlabeled data plus a limited number of labeled positive examples that are of interest to the users [65]. A study used the technique to develop an early warning system for adverse drug reactions (ADRs) [66], while another study used it to evaluate themes and emotions in a corpus of almost 60,000 RateMD reviews [67]. Table 1 summarizes recent research using several machine learning methods for topic classification.

Meanwhile, sentiment analysis, sometimes referred to as opinion mining, assists in determining the emotional context of free-text data. Sentiment analysis examines user expressions and connects emotions with them [31]. The analysis is advantageous for ascertaining how individuals feel about goods, activities, people, and services. Sentiment analysis has been applied in health care to assess patients’ perceptions of the quality of treatment they got [29,31]. Additionally, the English National Health Service [68] highlighted the importance of sentiment analysis data as a valuable and unique source of information for patients when selecting medical services [68]. The technique used by machine learning for sentiment analysis is similar to that taken for text classification. Sentiment analysis is frequently conducted using a supervised approach and includes some manual classification methods [48,51,52,53,55,56,57,58,62,69]. Even if the comments are pre-labeled, knowing what the negative and positive comments are particularly discussing takes reading through all of them. Moreover, the sentiment may be evaluated using unsupervised learning techniques such as LDA or lexicon-based libraries [12,61,63,64,67]. Additionally, several research used open-source or commercial sentiment analysis tools, such as TheySay [17], TextBlob [11], SentiWordNet [65], DICTION [59], TencentNLP [47], NVivo [25], and Keras [30]. Table 1 summarizes previous works on sentiment analysis using various machine learning methods.

### 2.4. Topics and Sentiments in Patient Online Reviews

Prior studies indicate that patient online reviews often address topics such as waiting times, healthcare system efficiency, and interpersonal quality [11,12,52,54]. However, other topics were identified as major issues, including communication, treatment efficacy and patient safety, the environment, and hospital costs [11,47,54,70].

Meanwhile, thorough analyses of patient online reviews showed that the majority of responses were positive [3,71]. An in-depth study using supervised learning discovered that patients who received a positive rating in Health Grades had a shorter wait time [27]. A similar study discovered that although empathy, friendliness, and explanation are often mentioned in positive sentiment, negative comments showed concerns regarding appointment access, appointment wait time, and time spent with a physician [52]. Additionally, a Facebook reviews analysis of hospitals in the United States discovered that waiting times, treatment efficacy, communication, diagnostic quality, environmental sanitation, and cost considerations are the factors most strongly associated with patients’ overall ratings [54]. Another study of patient feedback collected via Press Ganey questionnaires discovered that the most often used terms in positive patient responses are “nurse” and “doctor.” However, physical factors such as “Room,” reliability topics such as “discharge”, and responsiveness factors such as “tests and treatments” received the most unfavorable comments [30]. According to a study conducted on Chinese social media platforms, the predominant attitude about their healthcare is negative, with the doctor–patient relation category having the greatest percentage of negative sentiment, followed by service efficiency and nurse service [47]. However, both Chinese and American patients remarked on medical treatment, bedside manner, and appreciation/recommendation in their favorable evaluations, with Chinese patients focusing more on medical treatment and American patients focusing more on the recommendation. Additionally, Chinese patients’ evaluations of bedside manner focused more on physicians, while American patients’ reviews focused more on staff [61]. It is unsurprising that certain topics tended to be more negative than others. Discussions about time, money, or discomfort, for example, are unlikely to be positive [11].

Previous research using the LDA method discovered that the most frequently discussed subjects in patient online feedbacks were healthcare systems, interpersonal relationships, and technical elements [12,59,64]. Negative sentiment is often associated with personnel, timeliness, and diagnostic issues, while positive sentiment is strongly associated with interpersonal and technical excellence [59]. However, a study of Yelp reviews found that positive sentiment was linked with interpersonal quality and surgical treatment, whereas negative sentiment was associated with insurance, billing, and the cost of the hospital visit [12]. Another study used the SERVQUAL model and LDA to analyze NHS tweets and discovered that the dimensions of responsiveness and assurance are often discussed in negative sentiment, while sentiment ratings for empathy are entirely positive [64].

Although many prior studies have shown the percentage of subjects or themes with positive or negative sentiment, studies of patient online reviews should go beyond basic descriptive analysis and test theory-based hypotheses in order to offer additional clinical and policy implications [3]. In recent years, we have seen an increase in studies comparing patient online reviews and sentiments to traditional patient surveys [12,17,25,27,48,54,69], clinical outcomes [11], and hospital ranking [55]. Table 1 summarizes studies that demonstrate correlations between clinical outcomes, patient surveys, or other quality indicators, and the findings from machine learning/natural language processing analyses. However, the existing body of knowledge is still restricted due to a dearth of sophisticated statistical studies and their connection to additional quality indicators. A systematic review recommended doing more empirical research with relevant hypotheses, rigorous design, and data analytics on patient online reviews [3].

### 2.5. Proposed Work

Our proposed work was based on the aforementioned literature reviews. Given that social media continues to grow in all directions and penetrates virtually every sector in Malaysia and Southeast Asia, it is essential to use technology to improve healthcare services. Meanwhile, Facebook is a behemoth among social media sites. However, only minor research on machine learning and quality metrics utilizing Facebook data has been conducted [54,55,69]. Given Facebook’s popularity in Malaysia and its increasing use in healthcare, this research aims to close a gap by examining whether patient comments in Facebook reviews can be used in conjunction with patient satisfaction surveys and as a creative tool for assessing patient-perceived hospital quality of service. Additionally, most studies on patient online reviews have focused on populations in Western nations. Few studies have examined patient annotations among Chinese [47,61,63], Indian [55], and Korean populations [58]. Due to a lack of research involving Asian populations, we suggest that our proposed study adds value to patient online reviews from another Asian population through the Malaysian viewpoint.

Meanwhile, in terms of machine learning methods, our proposed study combines two approaches—topic classification and sentiment analysis—via the use of supervised learning. According to the research, conventional patient satisfaction surveys have a variety of disadvantages, and social media has been suggested as a possible alternative for assessing real-time patient satisfaction and mood. Additionally, a systematic review of the use of natural language processing (NLP) and machine learning (ML) to process and analyze patient experience data concluded that manual classification of free text comments remains the ‘gold standard’ method for analysis and is currently the only way to ensure that all pertinent patient comments are coded and analyzed [28]. Moreover, the study indicates that the patient inputs generated from free-text supplementing structured questionnaires are stable in nature, making them an attractive source of data for supervised learning. Numerous studies have used supervised machine learning to classify topics and sentiments [48,51,54,55,56,57,58]. Furthermore, we suggested that our machine learning topic classifier be trained using SERVQUAL dimensions. Few studies have assigned domains to classify themes in patient online reviews, such as SERVQUAL [64], CAHPS Dental Plan Survey [27], and HCAHPS [12]. The possible outcomes may be compared to conventional surveys of patient satisfaction or quality of care metrics. 

Another area of focus for the development of our own machine learning is that most software products and open-source tools used in topic or sentiment classification were originally designed to identify opinions about products in non-healthcare settings or other commercial industries or to be compatible with specific healthcare systems, particularly in Western countries [29]. Therefore, it may influence the accuracy and reliability of the classification in a range of healthcare settings. Additionally, commercial software is often expensive and unsuitable for long-term usage. Thus, our research demonstrated a novel approach for developing a new classifier and sentiment analyzer for service quality problems in Facebook reviews of a Malaysian public hospital.

In addition, our research should go beyond simple descriptive analysis and test theory-based hypotheses to provide additional clinical and policy implications. As such, we want to employ rigorous statistical methods such as regression analysis to ascertain the determinants of positive sentiment. Previous studies used analysis of variance (ANOVA) [27], Regression analysis [11,59,60,67,69], Pearson correlation [12,55], or Spearman’s rank correlation [25,55].

Furthermore, we want to compare patient online reviews with established quality measures in health care, such as the SERVQUAL, HCAHPS, hospital accreditation, and national quality indicators, among others. Previous research has discovered a moderate correlation between online patient feedback and the General Practice Patient Survey (GPPS) and the Friends and Family Test (FFT) [25]. Moreover, studies found several topics correspond to the CAHPS Dental Plan Survey [27] or HCAHPS survey [54]. Also, patients’ informal comments in Facebook help to predict the HCAHPS survey [69] while some topics in Yelp are correlated with positive or negative reviews but are not included in the HCAHPS [12]. However, sentiments in Twitter were not associated with the HCAHPS [11] and NHS inpatient survey [17]. Additionally, there were only weak to moderate associations between topics classified from NHS Choices comments and responses from the national inpatient survey [48]. Furthermore, by improving the sentiment score, one can bring their hospital ranking to the next level [55]. The findings may be utilized to improve the quality of hospital services and to offer more information to policymakers through online patient feedback in order to help them make more informed choices. Table 2 summarizes the proposed work in this research.

## 3. Materials and Methods

### 3.1. Hospital Facebook Data

Between January 2017 and December 2019, this study examined data from Facebook reviews that were publicly available on official public hospital Facebook pages. We used WebHarvy software (SysNucleus, Kochi, India) to gather all 3618 Facebook reviews from 48 official Facebook pages of Malaysian public hospitals. The automated parsing software was used in previous studies for web scrapping of online reviews [72] and extended to data mining [73]. The term “official” refers to the hospital Facebook page as one that had the hospital’s official name on the page, referenced the hospital’s official name in the page’s description, or connected directly to the hospital’s Facebook page from the hospital’s official website. We included only publicly accessible Facebook pages associated with the hospital, and all data gathered from the official Facebook page was retained in a pro forma checklist, such as the average number of stars the page had previously earned and the presence of complete hospital information on the page. The Facebook pages of hospital departments, as well as those of health organizations such as the Ministry of Health and the Institute of Medical Research, as well as those of non-governmental organization hospitals and long-term care facilities, were all excluded. All collected reviews were carefully screened, and any reviews that were deemed irrelevant due to company promotion or marketing were removed. These techniques of searching have also been used in earlier research [18,22,74]. All data was collected prior to the COVID-19 pandemic.

**Figure 1:**
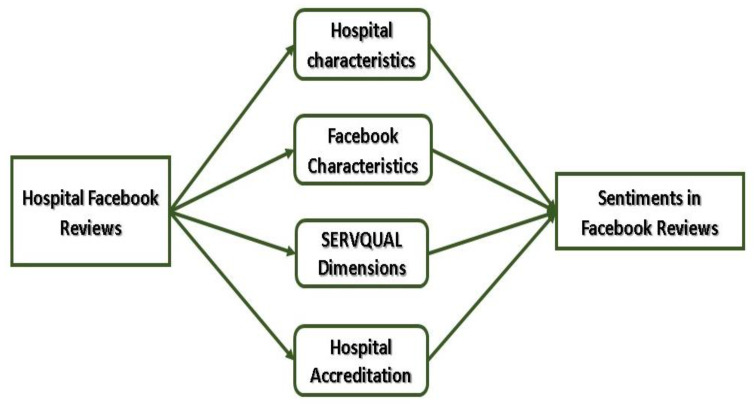
Conceptual framework for proposed work.

There are four major factors in patient online reviews that may influence sentiment in hospital Facebook reviews: hospital characteristics, Facebook characteristics, SERVQUAL dimensions, and hospital accreditation status. We quantified hospital characteristics by geographical region, urban or rural location, type of hospital (primary, secondary, or tertiary), and bed count. Additionally, factors pertaining to Facebook characteristics were examined, including previous Facebook star ratings, adequate hospital information on the hospital’s Facebook page, and whether or not the hospital responded to or reacted to patient comments in the Facebook reviews section. Moreover, Empathy, Assurance, Responsiveness, Reliability, and Tangible were the SERVQUAL dimensions evaluated in this research. Meanwhile, hospital accreditation refers to the status of accreditation conferred by the Malaysian Society for Quality in Health (MSQH) to public hospitals in Malaysia that met a wide range of hospital quality characteristics. The proposed work’s conceptual framework is shown in Figure 1.

Malaysia is a multicultural country with a rich linguistic and dialectal diversity. Malay is our national language, while English is our second language. As a consequence, we gathered reviews in those languages only. After standardizing the dual-language Facebook data, the Malay language data were translated manually by junior doctors into English for further study.

### 3.2. SERVQUAL Dimensions Classification

Through manual coding, a labeled data set was created to serve as a “gold standard” for machine learning quality dimension classifiers. The word “classifier” refers to the class labels applied during the human annotation step that is attempted to be correctly labeled by machine classification models [57]. The steps of topic classification were as follow:

1. Two hospital quality managers or SERVQUAL domain experts were appointed to do an initial “open” coding on batches of 100–300 Facebook reviews based on the MOH SERVQUAL patient satisfaction survey in order to create the source coding standard (Appendix A.1). Additionally, we supplemented descriptions in relevant dimensions using survey questions from previous SERVQUAL research.
2. Next, a randomly selected subsample of 300 Facebook reviews was used to assess intercoder reliability. The reliability subsample was coded independently by the raters. Cohen’s Kappa values were used to determine inter-rater agreement for each SERVQUAL dimension. The agreement between the coding of Tangible (Cohen’s = 0.885, *p* < 0.001), Empathy (Cohen’s = 0.875, *p* < 0.001), Reliability (Cohen’s = 0.736, *p* < 0.001), and Responsiveness (Cohen’s = 0.72, *p* < 0.001) characteristics from Facebook reviews was high, but agreement for Assurance (Cohen’s = 0.626, *p* < 0.001) was modest. Cohen’s coefficient averaged 0.769 across all dimensions.
3. Then, we utilized a sample of 900 manually labeled Facebook reviews to train our machine learning quality control tool.

**Figure 2:**
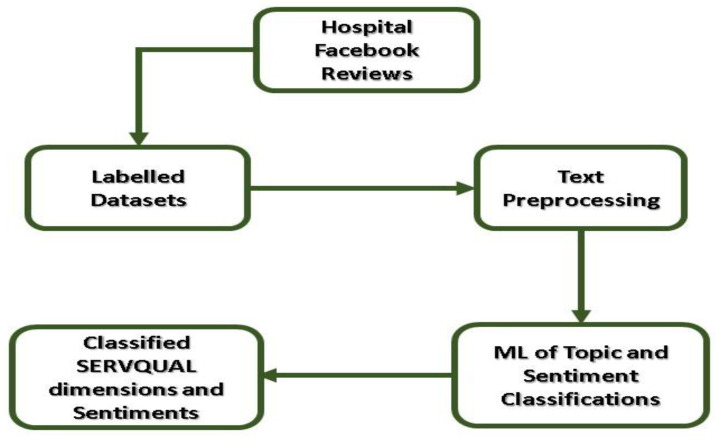
Machine learning development process.

The machine learning method analyses the properties of the individual phrases used in the Facebook reviews and utilizes this information to construct a topic classifier. To begin, the labeled dataset was pre-processed to remove URLs, numbers, punctuation marks, and stop words, as well as to reduce words to their simplest forms using a lemmatization method (e.g., treating as treat). Following that, we determined the weight of words using the term frequency-inverse document frequency (TF-IDF) method, which shows their importance to the documents and corpus. We next split randomly labeled data into 80% for training and 20% for testing using iterative stratification. For topic classification, a variety of multi-label classifier methods were trained, including Binary Relevance, Label Powerset, Chain Classifier, RAkEL: RAndom k-labELsets, MLkNN: Multi-label k-Nearest Neighbor, and BRkNN: Binary Relevance k-NN. We trained three basic classifiers for each technique: Naive Bayes (NB), Support Vector Machine (SVM), and Logistic Regression (LR). The NB, SVM, and LR classification techniques are all extensively used and have been shown to perform well on text classification problems [31,75]. The classifiers with multiple labels were assessed using Python’s scikit-multilearn package [76]. Several studies have used similar methods to build their topic categorization models in this investigation [11,51,52]. Figure 2 illustrates the process of topic classification. 

We used 5-fold cross-validation for evaluating the different classifiers. The classification models’ predictive performance scores varied between 0.13 and 0.25, suggesting that the models accurately categorized the reviews with an F1 value of 0.687 to 0.757. In general, when compared to other models and classifiers, the SVM model with chain classifier multilabel method has the highest accuracy (0.215) and F1-score (0.757). In addition, the hamming loss, which quantifies the percentage of erroneously predicted class labels, is more significant for topic classification models. In comparison to other models, the SVM model with chain classifier has the lowest hamming loss (0.273). As a consequence, the SVM model will be utilized to train the machine learning service quality classification, which will be trained using the Chain classifier method. The prediction performance of supervised machine learning with 5-fold cross-validation is summarized in Table 3, along with the accuracy ratings for the best classification model and multi-label classifier. 

### 3.3. Outcome: Sentiment in Facebook Reviews

The study’s conclusion is based on the positive or negative sentiments expressed in Facebook reviews. To evaluate the sentiment expressed in patient online reviews, human coding was used to generate a labeled data set that would serve as the “gold standard” for the machine learning sentiment analyzer. We enlisted the assistance of hospital quality managers familiar with patient satisfaction surveys to conduct open coding on 100–300 randomly selected Facebook reviews in order to generate a coding guideline (Appendix A.2). Following that, an intercoder reliability assessment was conducted using a randomly chosen subsample of 300 Facebook reviews. The agreement between the positive (Cohen’s = 0.721, *p* < 0.001) and negative (Cohen’s = 0.686, *p* < 0.001) sentiment coding was satisfactory. The neutral or unidentified category of review, on the other hand, had a lower degree of agreement (Cohen’s = 0.43, *p* = 0.027), which could be explained by the category’s more amorphous and heterogeneous nature. Thus, both quality managers will debate and re-evaluate the group of emotions that is neutral or unidentified. If the review remains neutral or unidentified, it will be deleted, since we prefer binary sentiment classification for reviews. Earlier research has validated and demonstrated that the binary technique outperforms multiclass sentiment classification (positive, negative, neutral) in terms of accuracy, recall, and F-score performance [56,77]. Following that, we labeled and pre-processed 1393 randomly chosen data instances in preparation for machine learning training. We divided the training set into 80% for machine learning training and 20% for testing the machine learning model using stratification. Our machine learning model was trained using the Python libraries nltk, spacy, and scikit-learn using three different types of classifiers: NB: Naive Bayes, SVM: Support Vector Machine, and LR: Logistic Regression. In this research, a few methods from prior studies were used to create a sentiment analyzer [48,51,62,77]. Our method of sentiment classification is shown in Figure 2.

Again, we used 5-fold cross-validation to evaluate the effectiveness of the machine learning sentiment analysis. SVM findings outperformed other machine learning methods in terms of accuracy (0.874), precision (0.903), and F1-score (0.919). However, naive Bayes has a greater recall than other algorithms (0.999). The assessment of the model after 5-fold cross-validation is summarized in Table 4. We selected the SVM model for our machine learning sentiment analyzer due to its excellent prediction accuracy.

### 3.4. Comparison with Hospital Accreditation

MSQH provided us a list of accredited public hospitals in 2018 and 2019. MSQH is a not-for-profit organization that was established in collaboration with the Malaysian Ministry of Health, the Malaysian Association of Private Hospitals, and the Malaysian Medical Association. MSQH criteria are applicable to all types of hospitals that are undergoing accreditation consideration, whether public or private, big, or small. Prior to the accreditation survey, a hospital pursuing accreditation must perform a self-assessment. The evaluation is carried out by a team of surveyors, who then analyze and vote on their findings by members of the Malaysian Council for Health Care Standards. During the study period, Malaysia had 69 accredited public hospitals.

### 3.5. Statistical Analysis

Due to the non-normal distribution of the data, numerical data were expressed as medians (interquartile range [IQR]) while categorical variables were expressed as frequencies and percentages in our statistical analysis. The connection between positive sentiments in Facebook reviews was determined using binary logistic regression analysis. The relationships were adjusted for hospital characteristics (region, bed count, urban or rural location, and type of hospital) and Facebook page characteristics such as previous star ratings, acceptable hospital information on the Facebook page, and administrator reaction in the Facebook review area. According to a prior study, these attributes are associated with positive sentiments [11]. We analyzed the results in terms of those that were statistically significant at *p*-value less than 0.05. All statistical test assumptions have been validated and met. The Hosmer–Lemeshow test, as well as the area under the receiver operating characteristic (ROC) curve, were utilized to validate the model fitness of our study. The data were analyzed using SPSS software version 26 (IBM Corp, Armonk, NY, USA).

## 4. Results

### 4.1. Hospital and Facebook Characteristics

Overall, 86 (63.7%) of Malaysia’s 135 public hospitals have an official Facebook account, with 48 (55.5%) allowing for customer input on the site. Twenty-five (52.08%) of the forty-eight hospitals that have Facebook reviews were accredited. Except for the western area, every region in Malaysia had at least ten hospitals that offered a Facebook review function: nationally, 37.5% of tertiary hospitals, 8.3% of secondary hospitals, and 54.2% of primary hospitals had Facebook review sections. Most of these hospitals were in urban areas and averaged 730 beds. Each hospital’s Facebook page received an average of 15.5 (27.5) reviews, with an average previous Facebook star rating of 5.00. (1.65). Numerous hospitals have contact details on their Facebook sites and have reacted to customer feedback.

### 4.2. Facebook Review Characteristics and Sentiment

We analyzed 1825 Facebook reviews in detail. Overall, the west (50.5%) and north (21.5%) areas received the bulk of evaluations. 87.2% of all reviews came from urban hospitals, while 88.8% came from tertiary institutions. Additionally, many evaluations (61.6%) were conducted in accredited hospitals, and the median number of beds was 730. In terms of prior Facebook ratings, the average was 4.70 stars. Most Facebook reviews provide sufficient hospital information on the hospital’s Facebook page but limited responses from the hospital administration. Most important, we had 1315 (72.1%) reviews with positive sentiment and 510 (27.9%) reviews of negative sentiment as identified by our machine learning sentiment analyzer.

### 4.3. SERVQUAL Dimensions

**Figure 3:**
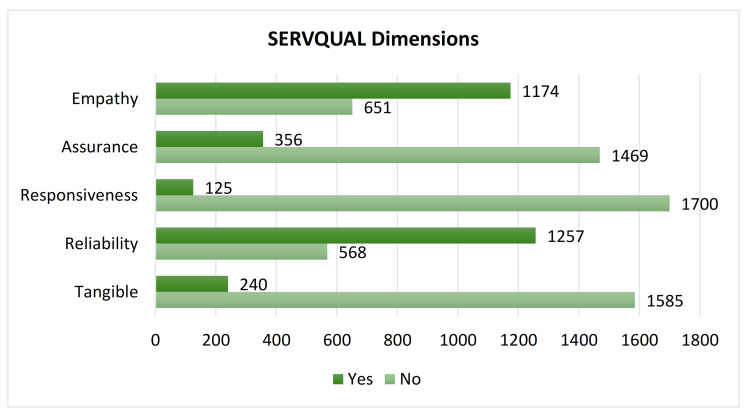
Overall SERVQUAL dimensions classified by machine learning.

Using a machine learning tool for SERVQUAL dimensions classification, overall, we had 240 (13.2%) reviews with tangible dimension, 1257 (68.9%) reviews of reliability, 125 (6.8%) reviews of responsiveness, 356 (19.5%) reviews of assurance, and 1174 (64.3%) reviews of empathy. The summary of overall SERVQUAL domains is presented in Figure 3. 

### 4.4. Determinants of Positive Sentiment

Univariate analysis of hospital characteristics revealed that 10.3% of positive reviews came from the east coast, 22.4% from the north, and 52.1% from the west. Each of the three areas (East coast, OR = 1.80 (95% CI: 1.34–2.86); North, OR = 2.11 (95% CI: 1.41–3.17); and West, OR = 2.03 (95% CI: 1.41–2.92)) is associated with positive sentiment. 1162 (88.4%) of positive reviews were from hospitals situated in urban areas, indicating a strong relationship between urban location and positive sentiment, with a 43% probability (95% CI: 1.07–1.92). Additionally, we discovered a significant relationship between previous Facebook star ratings and positive sentiment (OR = 1.09, (95% CI: 1.01–1.17)), but not with other Facebook features. The features of the hospital and Facebook are detailed in Table 5, and their relationship with positive sentiment is addressed in Table 6.

**Figure 4:**
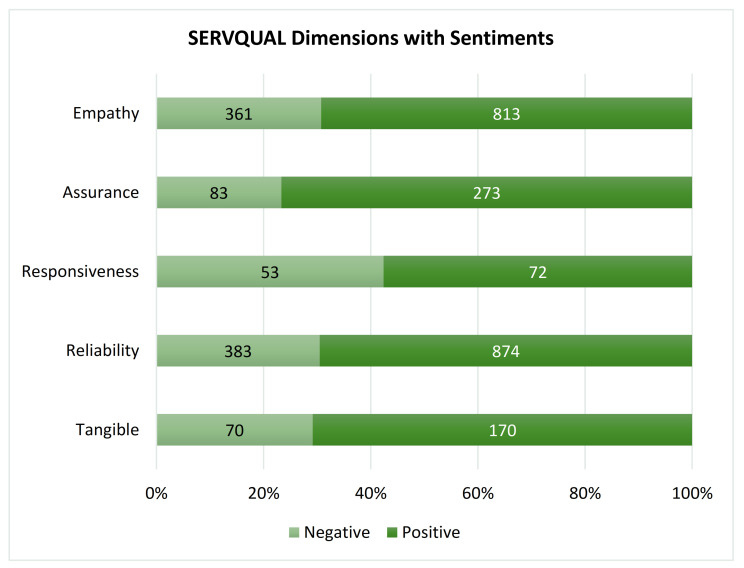
SERVQUAL dimensions with positive or negative sentiment.

On the other hand, 874 (66.5%) reviews were classified as reliability with a positive sentiment, 72 (5.5%) as responsiveness, 273 (20.8%) as assurance, 813 (61.8%) as empathy, and 170 (12.9%) as tangible with a positive sentiment. All SERVQUAL dimensions (Reliability, OR = 0.66 (95% CI: 0.52–0.83); Responsiveness, OR = 0.50 (95% CI: 0.35–0.72); Assurance, OR = 1.35 (95% CI: 1.03–1.77); and Empathy, OR = 0.67 (95% CI: 0.54–0.83)) were significantly associated with positive sentiment, except for the Tangible (OR = 0.93 (95% CI: 0.69–1.26)). Table 7 and Figure 4 summarize the proportion of SERVQUAL dimensions and sentiments, whereas Table 6 discusses their associations with positive sentiment.

In multivariable analysis, all significant variables or *p*-value less than 0.25 in the univariable analysis were selected in the process of model selection. We applied forward LR, backward LR, and manual selection methods using SPSS software to achieve a parsimonious model. The final model consisted of hospital location and SERVQUAL dimensions except for Tangible. A hospital located in an urban area has a 52% better chance of positive sentiment compared to a hospital in a rural area (95% CI: 1.12–2.04) when SERVQUAL dimensions were controlled. Moreover, assurance has 121% odds of positive sentiment (95% CI: 1.63–3.01) when other significant variables were adjusted. Meanwhile, with reliability, responsiveness, and empathy topics, the odds of having positive sentiment reduced by 58% (95% CI: 0.32–0.54), 51% (95% CI: 0.32–0.73), and 58% (95% CI: 0.33–0.55) respectively when location and other dimensions were controlled. The multivariate model has no interaction and multicollinearity in this study. The model was also acceptable as confirmed by the Hosmer–Lemeshow test (*p* = 0.648), 72.6% of Classification Table, and 62.3% of area under the Operating Curve (ROC) (*p* < 0.001). The multivariable analysis is described in Table 8. 

### 4.5. Association of Hospital Accreditation and Sentiment in Facebook Reviews

There were 824 (62.7%) positive Facebook reviews and 300 (58.8%) negative Facebook reviews from accredited hospitals. However, there was no significant relationship between hospital accreditation and positive sentiment (Crude OR = 1.18, (95% CI: 0.95–1.45), *p* = 0.131) or when hospital characteristics were adjusted for (Adjusted OR = 0.99, (95% CI: 0.73–1.34), *p* = 0.933). The details are in Table 5, and its univariate relationship with positive sentiment is presented in Table 6.

## 5. Discussion

To our knowledge, this is the first research to determine how patients evaluate the quality of hospital care and sentiment via the use of Facebook reviews in Malaysia and Southeast Asia. The study examined the hospital and Facebook characteristics of public hospitals, as well as SERVQUAL dimensions and sentiment analysis of Malaysian social media data. The research represents a critical first step in developing a technique for harnessing social media data, as well as an early effort to monitor public views of healthcare services via the use of a novel data source. Our findings indicate that social media use is increasing in Malaysia’s public hospitals, with the majority now having their own Facebook page. The findings confirmed research conducted in Taiwan, which established that the popularity of Facebook prompted healthcare institutions to create their own accounts on the site [78]. However, more than half of Malaysian hospitals’ Facebook sites lack a section dedicated to customer input. It is unknown if hospital officials disabled comments on purpose or were just unaware of the Facebook review feature.

### 5.1. Service Quality and Sentiment Analysis

This is the first study in Malaysia to develop a machine learning model for monitoring hospital quality. The findings of this study demonstrate how supervised machine learning algorithms may be used to accurately identify SERVQUAL dimensions and sentiment content in Malaysian Facebook reviews. Combining two elements of content analysis tasks, such as topic classification and sentiment analysis, is a novel technique, particularly in developing markets with a growing healthcare market and service provision such as Malaysia.

In terms of machine learning topic categorization, our research determined that the two most often discussed SERVQUAL dimensions were Reliability and Empathy. Previous studies indicate that waiting times, the efficiency of the healthcare system, and interpersonal quality are commonly discussed topics in patient online evaluations [11,12,52,54]. However, other topics have emerged as major issues, including communication, treatment effectiveness and patient safety, the environment, and hospital costs [11,47,54,70]. A systematic examination of patient internet evaluations corroborated the findings, revealing that these comments addressed the facility’s overall health care experience, including staff friendliness, empathy, time spent with patients, and wait time [3,34].

Meanwhile, our sentiment analysis revealed that the overwhelming majority of patient evaluations are positive. The generally favorable attitude on Facebook corroborates prior systematic reviews showing that social media users have a positive judgment bias [3,71]. However, other studies indicate that most social media comments are associated with negative feelings [30,47,75,79]. A comprehensive study of sentiment analysis in a social media platform for health care confirmed the contradictory findings of prevalent views [29]. Furthermore, additional systematic studies indicate that the polarity of sentiments was affected by the corpus- and thesaurus-based techniques employed in the research [28,31].

Except for the tangible dimension, our in-depth analysis revealed that all service quality themes were significantly associated with positive sentiment in this study. Our study’s sentiment evaluations found that reliability and empathy were highly valued. The outcome almost confirmed results from a study of NHS tweets conducted using the LDA method, which revealed their empathy is all positive, while their responsiveness and assurance were often criticized [64]. Additionally, our results corroborate previous research demonstrating a significant correlation between specific service quality topics mentioned in hospital-related social media comments and emotions [11,54]. Another study showed that patients who had a positive rating in Health Grades had a shorter wait time [27] whereas empathy, friendliness, and explanation are often mentioned in positive emotion [52]. Meanwhile, a Korean study found unfavorable sentiment about problems such as professionalism, competence, and treatment received via the use of a mixed conceptual model that included themes related to service quality [58].

Furthermore, a study showed that tangible, reliability, and responsiveness themes received more negative responses when utilizing Keras NLP software [30]. It was backed up by a large-scale analysis of China’s social media platforms using Tencent NLP, which discovered that the doctor–patient connection category had the greatest percentage of negative comments, followed by service efficiency and nursing care [47]. Despite the diversity of machine learning methods, it is unsurprising that certain subjects tended to be more negative than others—discussions about time, money, or discomfort, for example, are unlikely to be positive in patient online reviews.

Taken together, our findings suggest that Facebook review is a one-of-a-kind tool for engaging patients and eliciting hitherto untapped feedback. This study shows that these machine learning methods are more useful and informative than the general emotion-focused terms employed in traditional sentiment analysis. To improve the quality of the healthcare system, a systematic and effective approach is required. A paper calls for systematic, comprehensive monitoring and reporting of quality-improvement efforts, as well as a strong focus on reacting to and learning from events involving the quality of treatment [80]. To enhance healthcare outcomes in Malaysia, data on patient online assessments and systematic methods for analyzing patient input must be collected. The study’s approach allows policymakers to utilize public opinion about health care services on social media as a substitute for conducting and scheduling more costly national questionnaire polls. Additionally, because SERVQUAL serves as the foundation for public hospital patient satisfaction surveys in Malaysia, the conceptualization used in this study may be used in conjunction with the Ministry of Health’s hospital patient satisfaction survey and as a valuable early warning system for hospital quality management. Thus, we may determine societal views and integrate them into the design of high-quality healthcare services by systematically monitoring internet comments. Furthermore, we can help health care policymakers and providers in evaluating their quality of care in real-time and changing their policies or resources to better serve their patients [81,82].

### 5.2. Accreditation and Sentiment Analysis

Numerous previous studies established a correlation between social media results and clinical outcomes (e.g., mortality rate or readmission rate) [17,18,22,83] as well as with other structured quality measures such as HCAHPS, patient safety metrics, etc. [3,34]. Hospital accreditation in Malaysia attests to a hospital’s adherence to quality criteria, which includes treatment accessibility, appropriateness, effectiveness, and safety, as well as patient-centered activities, efficiency, and governance. The requirements place a premium on safety; an organization that fulfills all other criteria but falls short on safety will be refused accreditation [84]. After controlling for hospital factors, this study found no significant association between patient online sentiment and hospital accreditation. The result supports a previous study in Malaysia on hospital accreditation and online patient satisfaction [38]. Additionally, other study results indicated there was a weak or non-existent connection between clinical outcomes or indicators of quality of treatment [11,17,48]. The finding means that when compared to clinical results and quality metrics, sentiment in Facebook reviews should be evaluated with precaution. Because this research is still in its infancy with regards to the usage of Facebook data, robust techniques for comparing clinical outcomes or other quality criteria are required [3]. Our findings, however, suggest that there is some new data from social media that hospital administrators should closely monitor.

### 5.3. Implications/Recommendation

We suggest that each Malaysian public hospital create a separate or official Facebook page and monitor what their patients say on social media. By analyzing the emotion expressed in spontaneous tales, we may improve health care services by including factors that were previously unknown. Patient evaluations of health care services, for example, may help in identifying areas for service improvement, thus affecting health outcomes and use. In terms of public health efforts, patients’ views may assist health professionals in identifying potential obstacles to population-based interventions such as vaccination. Understanding how patients respond to different treatments may help in the creation of more tailored treatment regimens. Furthermore, patient evaluations show that patients agreed to their participation in online discussions. As such, health care administrators and policymakers must recognize that the findings are unlikely to be fully representative of the hospital service population. Rather than that, this examination of service quality problems should be seen in conjunction with conventional data collecting efforts. The study’s rapid identification and evaluation of certain service features are unique, and without it, healthcare organizations would have been unable to analyze massive amounts of real-time (unstructured) data.

### 5.4. Limitation and Future Scope

Numerous limitations exist in our study. To begin, although our study of Facebook reviews was prone to response and selection bias, this is also true of any conventional survey. We cannot rule out the potential of a causal relationship in our results due to the cross-sectional design of the study. Additional studies into the origins of these results would be beneficial. In addition, only 45 of 87 hospitals have Facebook reviews. Incorporating unofficial or unapproved Facebook sites for public hospitals may result in a change in public opinion. When it comes to sentiment analysis and topic classification, machine learning algorithms are only as effective as the training set used to train them. The primary limitation is that our dataset is deemed tiny in comparison to previous big data research, as social media reviews are still relatively new in Malaysia’s healthcare industry and our population is small. Malaysians’ use of social media, on the other hand, continues to increase year after year across all social demographic groups. As is the case in developed countries, we may anticipate a surge of social media evaluations of healthcare services. Another issue was the difficulty of manually coding social media information, especially for human coders with considerable expertise in quality management or the SERVQUAL model. This result is consistent with prior studies indicating ambiguity and a range of contextual perceptions in social media content as major issues [56,77]. Manual classification for supervised learning may become difficult as the quantity of comments on social media grows. To overcome this, a technique based on LDA may be used to discover numerous topics of discussion [85]. However, LDA has certain limitations of its own. It is expected that the produced topics are dependent on the sentiment distributions and that the generated words are conditional on the sentiment topic pairings. Thus, a weakly supervised joint sentiment-topic mode may be utilized to improve the accuracy of topic modeling by extending the maximum entropy discrimination latent Dirichlet allocation (MEDLDA) topic model [86]. 

Future research should focus on increasing sentiment analysis and topic classification performance, as well as on amassing a larger dataset of patient online evaluations, including those from the Malaysian private healthcare sector. Also, additional research is required to extend the method’s applicability to other types of free-text material on social media. For instance, different techniques may be added to strengthen the process, such as assessing unigrams, bigrams, or larger n-grams, as well as improving contextual polarity. Likewise, future research can be conducted using deep learning neural networks, such as DeepBlockScheme, a deep learning method based on blockchain technology [87], Kmean methods, a clustering algorithm for sentiment analysis [88], or graph convolutional networks (GCNs) and auxiliary node relations for modeling multi-target sentiment classification [89]. Moreover, to improve and ensure the security, confidentiality, and privacy of hospital data that was stored in the cloud, a blockchain-based secure storage architecture called BIIoVT can be implemented [90]. Furthermore, further studies are necessary to ascertain the connection between patient online reviews and other hospital quality measures. For example, evaluating the relationship between quality dimensions derived from social media reviews and patient satisfaction as measured by prior studies [35,70]. In addition, a comparison of the labeled dataset used in this study to other dictionaries or tools used in prior studies to enhance sentiment and text classification would be beneficial [28,29]. Further, future research may include other social media platforms (e.g., Twitter, Instagram, Tik-Tok, etc.) to provide health care practitioners and academics with a more complete picture of consumer views of healthcare quality of service. Finally, this research may be repeated to assess hospital service sentiment during the COVID-19 epidemic in Malaysia. 

## 6. Conclusions

We demonstrate how monitoring Facebook reviews with machine learning methods offers valuable, real-time data that is not available via conventional quality measures or surveys. According to this study, patients in Malaysia were generally satisfied with the services provided by public hospitals. With the exception of tangible, all SERVQUAL dimensions were significantly associated with positive sentiment. However, there is no association between hospital accreditation and the sentiment expressed in Facebook reviews. While many hospitals have their own Facebook pages and actively monitor them, we propose that hospital administrators and policymakers use this unique data stream to obtain a better knowledge of healthcare consumers’ experiences and the quality of care they receive. If an online review is strongly associated with a certain negative element of service quality, it suggests where hospital administrators should focus their efforts on patient care improvement.

## Figures and Tables

**Table 1:** Summary of Previous Studies.

				Topic Classification			Sentiment Analysis			
Study	Data Source	Population of Study	Number of Records	Supervised	Non-Supervised	Topics/Themes	Supervised	Non-Supervised	Other Tool	Associations. *
Lee et al, (2021) [64]	Twitter	UK	50,716		X	5	X	X		
Zaman et al, (2021) [54]	Facebook	USA	6581	X		7	X			X
Boylan et al (2020) [25]	NHS Choices	UK	1396			3			NVivo	X
Lin et al (2020) [27]	Health Grades	USA	204,751	X		17				X
Nawab et al, (2020) [30]	Press Ganey	USA	2830	X		13			Keras	
Hu et al (2019) [47]	WeChat, Qzone	China	29,017,055			9			TencentNLP	
Ko et al (2019) [60]	Vitals	USA	1,560,639		X	5				
Huppertz & Otto (2018) [69]	Facebook	USA	57,985				X			X
Abirami & Askarunisa, (2017) [55]	Multiple sources including Facebook, Twitter etc.	India	1941	X		5	X			X
Doing- Harris et al (2017) [52]	Press Ganey	USA	51,235	X	X	7/30	X			
Jimenez- Zafra et al (2017) [53]	Zorgkaart Nederland, Masquemedicos	Netherland, Spain	156,975 of COPOD & 743 of COPOS				X			
James et al (2017) [59]	RateMDs	USA	3712		X	6			Diction	
Hao et al (2017) [61]	RateMDs, Haodf	USA, China	156,558 of RateMD, 57,342 of Haodf		X	10		X		
Ranard el al (2016) [12]	Yelp	USA	16,862		X	50		X		X
Bahja & Lycett (2016) [62]	NHS Choice	UK	76,151		X	30	X			
Daniulaityte et al (2016) [56]	Twitter	USA	4000	X		3	X			
Hao & Zhang (2016) [63]	Haodf	China	731,264		X	10				
Hawkin et al (2016) [11]	Twitter	USA	11,602	X		10			TextBlob	X
Cole-Lewis et al (2015) [57]	Twitter	USA	17,098	X		10	X			
Jung et al (2015) [58]	Naver & Daum Web	South Korea	9450	X		6	X			
Rastegar-Mojarad et al (2015) [65]	Yelp	USA	6914	X*	X*	20			SentiWordNet	
Yang et al (2015) [66]	MedHelp	USA	3000	X*	X*	10		X		
Greaves et al (2014) [17]	Twitter	UK	1000	X		6			TheySay	X
Wallace et al (2014) [67]	RateMDs	USA	58,110	X*	X*	3		X		X
Greaves et al (2013) [48]	NHS Choice	UK	6412	X		3	X			X
Alemi et al (2012) [51]	RateMDs	USA	955	X		9	X			

* Associations with healthcare quality measures, patient surveys, hospital ranking, etc. COPOD = corpus of patient opinions in Dutch; COPOS = corpus of patient opinions in Spanish. X* = semi- or partial-supervised learning.

**Table 2:** Proposed work, its justification and comparison studies.

Proposed Work	Justification	Comparison Studies
Facebook as Data Source	Limited studies utilized Facebook data. Yet, Facebook is popular among patients and healthcare providers in Malaysia.	Studies that used Facebook data including Zaman et al. (2021) [54], Huppertz & Otto (2018) [69], and Abirami & Askarunisa, (2017) [55]
Asian as Study Population	Limited studies among Asian population	Chinese study by Hu et al. (2019) [47], Hao et al. (2017) [61] and Hao & Zhang (2016) [63], Indian study by Abirami & Askarunisa, (2017) [55], and Korean study by Jung et al. (2015) [58].
Topic and sentiment classification approach	Supervised learning via manual classification remains the ‘gold standard’ method for analyzing free text comments for patient online reviews.	Zaman et al. (2021), Abirami & Askarunisa, (2017), Daniulaityte et al. (2016) [56], Cole-Lewis et al. (2015) [57], Jung et al. (2015), Greaves et al. (2013) [48], and Alemi et al. (2012) [51] employed supervised learning for both topic and sentiment classifications.
SERVQUAL	Domains of a traditional survey of patient experiences (SERVQUAL) serve as a foundation for our ML topic classifier.	SERVQUAL by Lee et al. (2021) [64], CAHPS Dental Plan Survey by Lin et al. (2020) [27], and HCAHPS by Ranard et al. (2016) [12].
Advanced analytical approach	Most patient online review studies were descriptive. Hence, we aim to test the associations using advanced statistical analysis.	ANOVA by Lin et al. (2020), regression analysis by Zaman et al. (2021), Ko et al. (2019) [60], Huppertz & Otto (2018), James et al. (2017) [59], Wallace et al. (2014) [67] and Hawkin et al. (2016) [11], Pearson Correlation by Abirami & Askarunisa, (2017) and Ranald et al. (2017), Spearman’s rank correlation by Boylan et al. (2020) [25], Abirami & Askarunisa, (2017) and Greaves et al. (2014) [17].
Comparison with health care quality measures	Only a few studies compared standard health care quality measures such as HCAHPS, SERVQUAL, hospital accreditation or national quality indicators, etc.	GPPS and the FFT by Boylan et al. (2020), CAHPS Dental Plan Survey by Lin et al. (2020), HCAHPS survey by Zaman et al. (2021), Ranard et al. (2016), Huppertz & Otto (2018), and Hawkin et al. (2016), hospital ranking by Abirami & Askarunisa, (2017) and NHS inpatient survey by Greaves et al. (2014) and Greaves et al. (2013).

**Table 3:** Overall ML models performance.

Multilabel Classifier	Model	Accuracy	Recall	Precision	F1-Score	Hamming Loss
Binary Relevance	NB	0.147	0.761	0.701	0.730	0.315
	SVM	0.211	0.763	0.745	0.754	0.278
	LR	0.193	0.775	0.732	0.753	0.285
Label Powerset	NB	0.130	0.896	0.633	0.741	0.349
	SVM	0.166	0.799	0.679	0.734	0.323
	LR	0.158	0.825	0.669	0.739	0.326
Chain Classifier	NB	0.149	0.756	0.705	0.730	0.313
	SVM	0.215	0.761	0.753	0.757	0.273
	LR	0.191	0.770	0.727	0.748	0.290
RAkEL	NB	0.157	0.749	0.699	0.722	0.322
	SVM	0.186	0.764	0.724	0.743	0.295
	LR	0.180	0.765	0.726	0.745	0.293
MLkNN	N/A	0.140	0.737	0.697	0.715	0.327
BRkNN	N/A	0.157	0.648	0.732	0.687	0.330

NB = Naïve Bayes, SVM = Support Vector Machine, LR = Logistic Regression.

**Table 4:** Model evaluation of sentiment analyzer.

Model	Accuracy	Recall	Precision	F1-Score
NB	0.781	0.999	0.777	0.874
SVM	0.874	0.936	0.903	0.919
LR	0.843	0.992	0.833	0.906

NB = Naïve Bayes, SVM = Support Vector Machine, LR = Logistic Regression.

**Table 5:** Characteristics of Facebook reviews (*n* = 1825).

		Sentiment			
Variables		Negative		Positive	
		*n*	(%)	*n*	(%)
*Hospital Characteristics*					
Region	East Coast	53	(10.4)	136	(10.3)
	North	98	(19.2)	295	(22.4)
	West	237	(46.5)	685	(52.1)
	South	63	(12.4)	115	(8.7)
	East Malaysia	59	(11.6)	84	(6.4)
Location	Rural	81	(15.9)	153	(11.6)
	Urban	429	(84.1)	1162	(88.4)
Hospital Type	Primary	43	(8.4)	82	(6.2)
	Secondary	22	(4.3)	58	(4.4)
	Tertiary	445	(87.3)	1175	(89.4)
Beds (Median, IQR)		730	(604)	704	(563)
*Facebook Features*					
Admin Response	No	463	(90.8)	1188	(90.3)
	Yes	47	(9.2)	127	(9.7)
Adequate Hospital Information	No	35	(6.9)	76	(5.8)
	Yes	475	(93.1)	1239	(94.2)
					
*Hospital Accreditation*	No	210	(41.2)	491	(37.3)
	Yes	300	(58.8)	824	(62.7)

**Table 6:** Determinants of positive sentiment using univariate analysis (*n* = 1825).

Variables		Crude	95% CI	*p*-Value *
		OR	(Lower, Upper)	
*Hospital Features*				
Region	East Malaysia	Ref		
	East Coast	1.80	1.14, 2.86	0.012
	North	2.11	1.41, 3.17	<0.001
	West	2.03	1.41, 2.92	<0.001
	South	1.28	0.82, 2.02	0.282
Location of Hospital	Rural	Ref		
	Urban	1.43	1.07, 1.92	0.015
Type of Hospital	Primary	Ref		
	Secondary	1.38	0.75, 2.56	0.301
	Tertiary	1.39	0.94, 2.03	0.097
Numbers of Bed		1.00	1.00, 1.00	0.017
*Facebook Features*				
Admin Response to Review	No	Ref		
	Yes	1.05	0.74, 1.50	0.773
Adequate Hosp Info	No	Ref		
	Yes	1.20	0.79, 1.82	0.385
Previous Facebook Star Ratings		1.09	1.01, 1.17	0.033
*SERVQUAL*				
Tangible	No	Ref		
	Yes	0.93	0.69, 1.26	0.651
Reliability	No	Ref		
	Yes	0.66	0.52, 0.83	<0.001
Responsiveness	No	Ref		
	Yes	0.50	0.35, 0.72	<0.001
Assurance	No	Ref		
	Yes	1.39	1.03, 1.77	0.030
Empathy	No	Ref		
	Yes	0.67	0.54, 0.83	<0.001
*Hospital Accreditation*	No	Ref		
	Yes	1.18	0.95, 1.45	0.131

* Simple Logistic Regression.

**Table 7:** SERVQUAL dimensions in Facebook reviews (*n* = 1825).

				Sentiment			
Variables		Overall		Negative		Positive	
		*n*	(%)	*n*	(%)	*n*	(%)
Tangible							
	No	1585	(86.8)	440	(86.3)	1145	(87.1)
	Yes	240	(13.2)	70	(13.7)	170	(12.9)
Reliability							
	No	568	(31.1)	127	(24.9)	441	(33.5)
	Yes	1257	(68.9)	383	(75.1)	874	(66.5)
Responsiveness							
	No	1700	(93.2)	457	(89.6)	1243	(94.5)
	Yes	125	(6.8)	53	(10.4	72	(5.5)
Assurance							
	No	1469	(80.5)	427	(83.7)	1042	(79.2)
	Yes	356	(19.5)	83	(16.3)	273	(20.8)
Empathy							
	No	651	(35.7)	149	(29.2)	502	(38.2)
	Yes	1174	(64.3)	361	(70.8)	813	(61.8)

**Table 8:** Determinants of positive sentiment using multivariate analysis (*n* = 1825).

Variable		Adjusted OR	95% CI (Lower, Upper)	*p*-Value *
Location	Rural	Ref		
	Urban	1.52	1.12, 2.04	0.007
Reliability	No	Ref		
	Yes	0.42	0.32, 0.54	<0.001
Responsive	No	Ref		
	Yes	0.49	0.32, 0.73	0.001
Assurance	No	Ref		
	Yes	2.21	1.63, 3.01	<0.001
Empathy	No	Ref		
	Yes	0.42	0.33, 0.55	<0.001

* Multiple Logistic Regression. Constant = 1.686. Forward LR, Backward LR, and Manual selection were applied. No significant interaction or multicollinearity. Hosmer–Lemeshow test = 0.648. Classification Table = 72.6%. Area under the operating curve (ROC) = 62.3% (*p* < 0.001).

## Data Availability

The Facebook data presented in this study are available on request from the corresponding author. The data are not publicly available due to privacy and confidentiality. However, restrictions apply to the availability of hospital data. Data was obtained from MSQH and Ministry of Health and are available from the authors with the permission of both organizations.

## Appendix A

### Appendix A.1. SERVQUAL Guideline

**Domain**	**Description**	**Facebook Reviews Example**
Tangible	General: The appearance of employees, equipment, and physical facilities of the hospital. Specific: The hospitals have up to date equipment. The physical facilities are visually new or outdated. The staff are well dressed, appear neat and good looking. The appearance of the physical facilities of the hospital are well maintained with the type of services provided.	“Cleanliness of the Hospital is good” “Car parking is difficult and limited” “Satisfied with the facilities. Large room, feels like a hotel.” “The hospital is well maintained, and their food is delicious.”
Reliability	General: Accurate, dependable, and consistent performance of the service. Specific: When the hospital promised to do something by a certain time, it does so. Hospital service is efficient and dependable. The hospital provides services at the time as promise to do so. The hospital keeps the records accurately or at online.	“My appointment scheduled at 9 a.m. but then it was postponed to 12.00 p.m. Unbelievable.” “System needs to be improved especially discharge process. It took hours to settle it.” “Efficient and top-quality hospital services” “Staff mistakenly collected medical record of other patient with similar name of mine”
Responsiveness	General: Willingness to provide prompt service to the patients. Specific: The hospital let patients know exactly when the services will be performed. The staff give prompt services to patients upon request. The staff are always willing to help their patients. The staff give medical attention promptly.	“My specialist took his time to explain me about my disease and how he will treat it” “They answered all my questions during the admission.” “Arrived at emergency department due to road traffic accident and the medical team immediately respond to it.” “I don’t feel any pain throughout the minor surgery on my arm, and it was done in a flash”
Assurance	General: the staff knowledge and courtesy, ability to inspire trust, confidence, and security; also reflects on confidentiality and privacy of patients. Specific: The staff are trustworthy. Patients feel safe in their transactions with the hospitals. The staff are polite, friendly. The staff have adequate support from the hospitals to do their jobs well.	“The surgery was successful. Mr A is a competent and trusted surgeon.” “I feel comfortable and safe in this hospital. Just like at home” “The staff at the front desk was rude.” “The doctors and staff nurses in this hospital are skillful and well-trained”
Empathy	General: Providing convenient services and giving attention or patience of the staff to the patients’ needs. Specific: The staff give patient personal attention and helpful. The staff are knowledgeable to understand patient’s specific needs. The hospital has patient best interests at heart. The hospital has operating hours convenient to all the patients. Cost of treatment is affordable for patients	“Nurses are very helpful.” “A staff came and offered to help my father climb stairs without we ask him. We appreciated his kindness.” “They are very concerned about patient’s condition and served it with their heart” “The price is affordable compared to private hospital.”

### Appendix A.2. Sentiment Analysis Guideline

**Category**	**Description**	**Facebook Reviews Example**
Positive	Expression of liking, approval, gratefulness (Like, love, support, thankful, etc.)	“I like this hospital. Doctors and nurses are pleasant and helpful.” “Thank you for your service, Doctor and nurses.”
	Positive qualities of hospital services and facilities (Clean room, efficient, fast appointment, affordable, etc.)	“The wait time was brief. The pharmacy counter did an excellent job.” “The room is neat and tidy, and the food is delicious. I really like it.”
	Positive qualities of staff (Polite, friendly, helpful, responsive, etc.)	“Staff are polite and kind.” “Dr. B took her time explaining my health condition until I understood it. It was greatly appreciated.”
	Encourage or recommend others to use	“I recommend having your baby delivered at this hospital.” “I like their antenatal counselling and will recommend it to other couples. It is extremely beneficial to us.”
	Positive/desirable effects of service (Successful treatment/procedures, good health outcome, etc.)	“I’d like to thank Mr A for performing bowel surgery on my father. He is now doing well.” “I found the physiotherapy session to be beneficial. I’m able to walk with less pain now.”
Negative	Expression of disliking or disapproval (Do not like, hate, etc.)	“I hate the security guard.” He was impolite to me!” “I’m not a fan of the food service here. The food has no taste.”
	Negative characteristic of hospital services or facilities (Poor maintenance, slow service, expensive, long waiting time, etc.)	“The discharge procedure was extremely slow.” “There are a limited number of parking spaces available, and getting one is difficult.” “We waited for 5 h at the out-patient clinic before seeing the doctor. This is intolerable.”
	Negative qualities of staff (Rude, not-friendly, not-helpful, slow responsive, incompetency, etc.)	“Staff nurses were rude and stubborn. I requested assistance but received no response.” “The doctor criticised us for arriving at the emergency department at 3 a.m. for treatment. We were annoyed by his attitude.”
	Negative/undesirable effects (Surgical or procedural complications, medicolegal, poor health outcome, etc.)	“My father fell in the toilet and was left alone for a few minutes. The hospital director must explain the incident to our family.” “After being admitted to this hospital two days ago, my husband’s condition has deteriorated. No one, however, can explain the situation to us”.
Neutral	Review that reports factual information/no opinion.	“Serdang Hospital is one of the Klang Valley’s cardiac centres”. “A Muslim-friendly hospital”
	Review as questions	“Do you have any spine surgeon in your hospital?” “How to get an appointment with your ear. Nose and throat (ENT) clinic?”
	Too ambiguous/unclear/greetings only	“Good morning.” “No comment.” “Let’s wait and see first”
